# Accelerated Genomic Evolution and Divergence of *Escherichia coli* Under Phage Infection Stress: Emphasizing the Role of IS Elements in Changing Genetic Structure

**DOI:** 10.3390/microorganisms14010160

**Published:** 2026-01-11

**Authors:** Shuyang Wen, Lihong Yuan, Yingying Li, Jiayue Yin, Peng Luo

**Affiliations:** 1 School of Life Sciences and Biopharmaceutics, Guangdong Pharmaceutical University, Guangzhou 510006, China; 2 CAS Key Laboratory of Tropical Marine Bio-Resources and Ecology (LMB), Guangdong Provincial Key Laboratory of Applied Marine Biology (LAMB), South China Sea Institute of Oceanology, Chinese Academy of Sciences, Guangzhou 510301, China; 3 University of Chinese Academy of Sciences, Beijing 101408, China

**Keywords:** high-throughput sequencing, *Escherichia coli*, phage infection stress, genomic evolution, IS elements

## Abstract

The phage-resistant mutant (PRM) strains of *Escherichia coli* (*E. coli*) exhibited abundant genetic and phenotypic diversity. IS elements played a vital role in creating various genetic divergences and regulating gene functions under phage infection stress. Genetic variations of PRM strains derived from *E. coli* MG1655 and mutation frequencies of coevolved *E. coli* populations with phages were explored by high-throughput sequencing and resequencing. Infrequent-restriction-site PCR (IRS-PCR) and carbon utilization test revealed the genetic and phenotypic diversity of the PRM strains. Numerous and discrepant mutation sites (MSs) were observed in the PRM strains and the coevolved populations, and many MSs were related to the synthesis of flagella and LPS, which often serve as receptors in a phage invasion. The insertions of various IS elements in key gene locations were also frequently found in the PRM strains, which indicate for the first time that IS elements played a vital role in generating genetic divergence and regulating gene functions under phage infection stress. Resequencing revealed that the coevolved populations at three evolving stages had discrepant profiles of MSs, and nearly all detected MSs occurred in the coevolved populations, which led to coexisting phages that increased the mutation rates and expedited the occurrence of the defective MSs in *E. coli* populations. In summary, our results reveal that the widespread and abundant presence of phages may provide one important force driving bacterial genomic evolution and prompt bacterial genetic divergence via accelerated mutation and increased mutation rates in the *E. coli* genome.

## 1. Introduction

Bacteriophages (phages) are the most abundant and genetically highly diverse organisms on Earth [1,2,3]. Phages propagate through hijacking the replication and metabolic mechanisms and inducing the lysis of bacterial hosts, and thus, provide very strong selection stress for bacterial survival and evolution [4,5,6], resulting in phage-resistant bacterial populations. Likewise, phages can evolve diversified mechanisms to counter-adapt against phage-resistant bacteria [6,7,8]. For a considerable period, it has been widely recognized that the interaction between bacteria and bacteriophages serves as an important driver of their ecological and evolutionary processes [9,10,11,12]; however, studies on the genetic variations in phage-resistant mutant (PRM) strains and the genomic evolution of coevolved bacterial populations with phages based on whole-genomic data are inadequate. Several studies on the comparative genomics between PRM and ancestor strains indicate that in most cases, genetic mutations are limited to a low number of point mutations and insertions/deletions (InDels) of small fragments, which mainly affect the expression of cell surface structure genes, such as LPS [13,14,15,16,17,18]. Though some researchers have revealed that the antagonistic interaction between phages and bacterial hosts drives genome-wide host evolution and increases mutation rates, these studies were mainly based on a quantitative comparison of mutation rates and genotyping of a few strains from coevolved and evolved bacterial populations [19,20,21,22]. Little is known about the genetic variation and evolutionary characteristics of bacterial genomes under phage infection stress. The rapid development of high-throughput complete-genome sequencing allows us to analyze these genetic variations at the whole-genome scale. Furthermore, the resequencing technique provides a new approach to probe into the occurrence frequencies and profiles of mutation sites in one biotic population. However, so far, this technique used to investigate the occurrence frequencies of mutation sites from the co-evolution of phage φ2 and *Pseudomonas fluorescens* [23].

*Escherichia coli* (*E. coli*) is the most studied prokaryotic model organism and is one of the most important bacterial species in the fields of biotechnology and microbiology. Though resistance to lytic phages has been noticed in mutant *E. coli* strains for over 70 years [24], the genetic variation and evolutionary characteristics of *E. coli* under phage stress remain poorly characterized. In this study, we first isolated numerous PRM strains derived from the ancestor *E. coli* MG1655 under phage infection stress. The whole genomic sequences of several typical genetically divergent mutants were analyzed by focusing on mutation sites (MSs) and their potential functions. Moreover, genome resequencing of the evolved populations of *E. coli* MG1655 and the coevolved populations with enriched and mixed phages during different periods was carried out to characterize the mutation sites and their occurrence frequencies. Numerous and diverse MSs in the PRM strains and discrepant profiles of MSs between the evolved and coevolved *E. coli* populations suggest that the varied and abundant presence of phages may provide one important force driving bacterial genomic evolution and accelerate bacterial genetic divergence via an increased mutation rate. In these processes, insertion sequence (IS) elements revealed a changed genetic structure and regulated gene expression in *E. coli* PRM strains and coevolved *E. coli* populations for the first time.

## 2. Materials and Methods

### 2.1. Enrichment of Mixed Phages Infecting E. coli and Isolation of PRM E. coli Strains

Mixed phages capable of infecting *E. coli* MG1655 were enriched from sewage samples collected from the Pearl River in Guangzhou, China, using the method described by Luo [25]. The double-layer plate method was used to isolate PRM colonies [26]. Each 0.5 mL culture of *E. coli* with OD_600nm_ = 0.6 was mixed with 0.5 mL of mixed phage fluid at an approximative multiplicity of infection (MOI) of 30, added to 4 mL LB broth (supplemented with 0.7% agar) preheated at 45 °C, and then immediately poured onto a lower LB agar plate. The plates were incubated overnight at 37 °C, and then PRM colonies with different morphologies were selected and purified four times.

### 2.2. Genotyping of Phage-Resistant Isolates Using IRS-PCR

The digestion and ligation of template DNA were carried out as described previously [27]. The restriction endonuclease enzymes *HhaI* (TaKaRa) and *XbaI* (TaKaRa) were used in this study. The decomposition and connection operations of the template DNA and the PCR process were carried out according to the previously described methods [28,29]. The PCR products were loaded into wells of a 8% polyacrylamide gel prepared from a 30% acrylamide–bisacrylamide (29:1) solution in 1× TBE buffer (0.045 M Tris-borate, 0.001 M EDTA). After electrophoreses for 5 h at 200 V, the gel was stained with ethidium bromide (0.5 mg/mL) for 30–45 min, destained in water for 25 min, and photographed with UV illumination. IRS-PCR served as preliminary screening for divergent mutant strains.

### 2.3. Analysis of Biochemical Profiles of Selected E. coli PRM Strains

The utilization of 71 carbon sources and the sensitivity to 23 chemical substrates of the PRM strains screened by IRS-PCR were assayed using GEN III microplates (BIOLOG, Hayward, CA, USA). The cells of tested strains were suspended in inoculating fluids, and 100 μL aliquots of suspended cells were added to the microplates. After 24–36 h of incubation, the microplates were analyzed using a Biolog Microstation System.

### 2.4. Whole-Genome Sequencing of the Selected PRM Strains

The genomes of the selected PRM strains were de novo sequenced using the Illumina HiSeq 2500 platform (GENEWIZ, Suzhou, China). Reads were assembled according to quality control results using velvet (v1.2.10) [30,31] and gap-filled with SSPACE (v3.0) [32] and GapFiller (v1–10) [33].

The genome comparison between the PRM strains and ancestor *E. coli* MG1655 was carried out using MAUVE (v2.4.0) [34] software. The coding genes were annotated using BLAST in NCBI (https://blast.ncbi.nlm.nih.gov/Blast.cgi). The GO (Gene Ontology) [35] and KEGG (Kyoto Encyclopedia of Genes and Genomes) [36] databases were used to annotate the functions of genes and pathways, respectively.

### 2.5. PCR Detection of Genetic Changes in Gaps Between Scaffolds, Followed by Sequencing

PCR primer pairs based on the genome sequence of *E. coli* MG1655 were designed to amplify the gaps that spanned two adjacent scaffolds in the PRM strains. Only the PCR products of the PRM strains that had obviously different sizes from those of ancestor *E. coli* MG1655 were purified for subsequent sequencing.

### 2.6. Evolved and Coevolved Samples and Resequencing

5 mL of *E. coli* MG1655 was cultured to the early exponential phase (OD_600nm_ = 0.6–1), and labeled as sample I0. At the same time, inoculate 60 μL of *E. coli* MG1655 culture fluid into 5 mL LB broth, with 40 replicates. Among them, 20 replicates were inoculated with 20 μL of phage fluid at a multiplicity of infection (MOI) about 10 to serve as coevolved populations, wherein bacterial cells coevolved with enriched phages, and the other 20 replicates were inoculated with 20 μL of LB broth to serve as isolated evolved populations. They were incubated at 37 °C in a shaker. When the cultures’ OD_600nm_ values reached approximately 0.4, 1.0, and 1.5, 5 replicates inoculated with phage were mixed and labeled as samples E1, E2, and E3; meanwhile, 5 replicates inoculated with LB were mixed and labeled as samples C1, C2, and C3. The average times taken for the OD_600nm_ values to reach 0.4, 1.0, and 1.5 in the E1, E2, and E3 groups were 21 h, 27 h, and 33 h, respectively. The cells of all the samples were centrifuged and collected for DNA extraction.

Genome resequencing of *E. coli* cell populations from all the samples was carried out by Illumina HiSeq X Ten. The pass filter data allowed for removing adaptors and bases of low quality using Cutadapt (version 1.9.1) to obtain clean data for continuous data analysis. The alignment software BWA (version 0.7.12) was used to map clean data to the reference genome of *E. coli* MG1655 (GenBank No: NC_000913.3). The detection of SNVs (single-base variations) and InDels (insertion/deletion mutations) was performed using Samtools (version 1.1) and the Unified Genotyper module of GATK (version 3.4.6) software. Annotation for the SNVs/InDels was performed using Annovar (version 21 Feb 2013). Furthermore, InDels were detected and their frequencies were calculated no matter how many bases they contained. To ensure the accuracy of the resequencing data, only mutation frequencies > 10% were counted.

## 3. Results

### 3.1. IRS-PCR Exhibited Extensive Genetic Variations in E. coli PRM Strains

**Figure 1:**
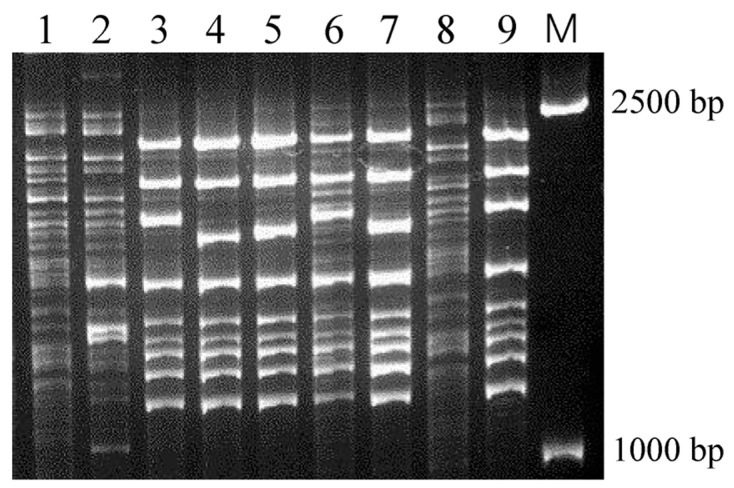
**Genotyping of typical *E. coli* PRM strains using IRS-PCR.** M: DNA marker; 1: *E. coli* MG1655; 2: PRM strain 008; 3: PRM strain 025 (MGM01); 4: PRM strain 039 (MGM02); 5: PRM strain 047 (MGM03); 6: PRM strain 052 (MGM04); 7: PRM strain 068 (MGM05); 8: PRM strain 083; 9: PRM strain 094 (MGM06). Six PRM strains showed obviously different DNA fingerprints (featured by bright and unique bands) from ancestor strain MG1655, and they were renamed as MGM01–MGM06.

After co-culturing Escherichia coli MG1655 with a mixture of phages for 20 h, PRM strains were collected and purified. In total, 101 PRM strains purified from the resistant mutant colonies were obtained. To determine the genetic variations in the PRM strains, genotypes of the 101 PRM strains were assayed using conventional infrequent restriction site PCR (IRS-PCR), a DNA-based fingerprinting method used to produce strain-specific electrophoretic patterns for genotyping [37]. Six PRM strains exhibited obvious genetic variations and they were renamed MGM01–MGM06 (Figure 1). The sizes of the altered DNA bands mainly ranged between 1000 and 2500 bp, and they were brighter than those that remained unaltered. Six PRM strains had several identical bands, which suggested that they likely generated similar genetic changes in some chromosomal sites.

### 3.2. Biolog Assays Revealed Phenotypic Diversity of Six E. coli PRM Strains

**Figure 2:**
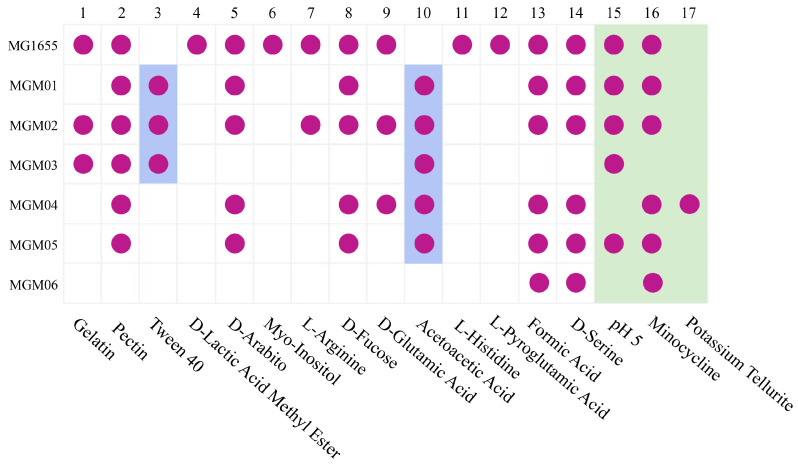
**Biochemical profiles of *E. coli* MG1655 and PRM strains revealed using Biolog assays.** Totally, 17 discrepant reactions among six PRM strains and strain MG1655 were observed. The purple circles in boxes represent a positive reaction. The blue background represents some PRM strains obtained the ability of utilizing Tween 40 and Acetoacetic Acid. The boxes with light green background represent chemical sensitivity.

The phenotypic variations in the six PRM strains (MGM01–MGM06) were further assayed using GEN III microplates (BIOLOG, USA). The Biolog assays showed some phenotypic discrepancies between the six PRM strains, which had 17 differentiated reactions compared with the primary strain MG1655, among which 14 were related to carbon utilization and 3 were related to chemical sensitivity (Figure 2). The reaction spectrum of each PRM strain is unique, but all PRM strains tended to lose more phenotypes than they acquired. Among them, MGM06 lost 11 positive phenotypes (including losing the ability to utilize 10 carbon sources) compared with MG1655. All the PRM strains lost the ability to utilize D-Arabitol, myo-Inositol, L-Histidine, and L-Pyroglutamic Acid. MGM04 and MGM06 became sensitive to low pH (pH 5), and MGM03 became sensitive to minocycline. In contrast, MGM04 lost the sensitivity to Potassium Tellurite. The Biolog assays have shown that 6 PRM strains have rich phenotypic changes; however, these changes showed no significant associated with anti-phage infection.

### 3.3. Numerous Scattered Mutations Occurred Within Coding Genes in Six PRM Strains Revealed Using Comparative Genomics

To explore the genetic variations in six PRM strains, their draft genome sequences were acquired and comparative genomics of *E. coli* MG1655 and six PRM strains was carried out. The comparative genomics revealed 12 scattered non-synonymous single-point mutations (SPMs) and one InDel in the six PRM strains; specifically, MGM01–MGM06 contained 1, 5, 1, 3, 5, and 3 SPMs, respectively, and each PRM strain had different combinations of MSs. A total of 13 MSs covered 11 genes that encode proteins with different functions, including IS translocation, LPS synthesis, lipoprotein synthesis, osmotic regulation, DNA replication, and recombination (Table 1). Some mutations occurred at the same gene sites in two or more strains, such as *insB-4*, *ydfK*, and *opgH* (Table 1); among them, the nonsense mutation in *opgH* caused the premature termination of protein biosynthesis. *ydfK* and *waaJ* contained two MSs each (one nonsense mutation in *waaJ*). Among the six PRM strains, MGM05 showed the most unique MSs, including a 7 bp insertion in the DNA-packaging gene *nohA*, causing a frameshift mutation (Table 1).

### 3.4. Concentrated SPM Sites and Large InDels Occurred in the Genomes of Six PRM Strains

**Figure 3:**
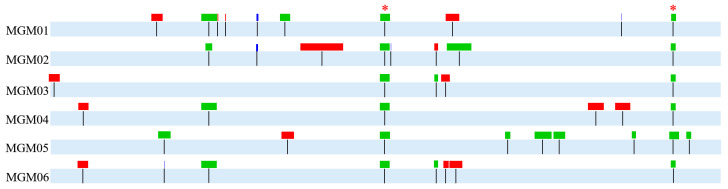
**Schematic map of concentrated SPM sites and large InDels in the genomes of *E. coli* PRM strains.** Vertical lines represent the locations of concentrated SPM sites and large InDels. Red boxes represent large insertions, green boxes represent large deletions, and blue boxes represent concentrated SPM sites. The length of boxes proportionally represents the sizes of concentrated SPM sites and large InDels. “*” represents two nearly identical deletion sites shared by six PRM strains.

In addition to the scattered SPM sites and one InDel, the six PRM strains harbored 46 variant regions containing concentrated SPM sites (SPMs > 5 within the range of 300 bp) and large InDels (≥10 bp) (Figure 3), among which 12 variant regions occurred in the non-coding DNA tandem repeats (TRs), 7 spanned the coding genes and upstream/downstream TRs, 11 occurred within coding genes, 8 adjoined rDNAs or tDNAs, and 8 rarely occurred within rDNA and tDNA (Table S1). Each PRM strain had a different profile of concentrated SPM sites and large InDels. MGM01 contained the most concentrated SPM sites and large InDels, while MGM03 harbored the least.

Frequent mutations [38] were observed in rRNA (including 5S, 16S, and 23S) and tRNA genes or their related DNA regions, accounting for 34.7% of the total concentrated SPM sites and large InDels. The deletion of three consecutive tRNA-Args in MGM02 was the largest detected deletion. Moreover, mutations that occurred in TR regions were mainly caused by the expansion or contraction of core sequences. The six PRM strains shared two nearly identical deletions of TR regions, which demonstrated that the prevalence of TRs caused mutations in the PRM strains. Furthermore, the genes containing mutations were significantly different between the six PRM strains. The mutated genes encode proteins involving various functions, including inner membrane protein synthesis, protein modification, amino acid transport, IS translocation, periplasmic sensing, bacterial toxin, and DNA recombination. It is worth noting that some mutated genes contained abundant repeat sequences, such as the RHS element genes *rhsC* and *rhsA* and the RHS-like gene *ybfO*.

### 3.5. One Big Deletion and Frequent Insertions of IS Elements Were Identified in PRM Strains Using Gap-Filling PCR

**Figure 4:**
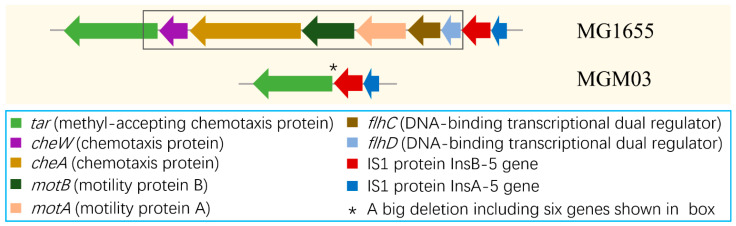
**Schematic location of the big deletion of 6068 bp (including six genes) upstream of an IS1 element in MGM03.** Genes are represented as arrows (the length of the arrows corresponds to the length of the genes). The deletion fragment is shown in a box. Functions of the genes with different colors are also listed in the figure.

Under the same sequencing conditions, the locations of gaps between two adjacent scaffolds varied in the six PRM strains, which implied that some genetic variations hid between these gaps. Therefore, we manually filled the gaps using PCR, where only PCR products with different sizes were screened out for sequencing. Consequently, one big deletion and eight insertions were identified in six mutant strains. MGM03 harbored a big deletion of 6068 bp (from nt1972435–nt1978502) covering seven genes: *tar*, *cheW*, *cheA*, *motB*, *motA*, *flhC*, and *flhD* (Figure 4). The deletion not only eliminated two intact operons containing *cheW*, *cheA*, *motB*, *motA*, *flhC*, *and flhD*, which participate in bacterial chemotaxis, motility, and flagellar synthesis, but also destroyed the transcription of another upstream operon containing *tar* and five genes. Notably, the series of deleted genes located upstream of an IS1 element comprised *insA-5* and *insB-5* (Figure 4).

**Figure 5:**
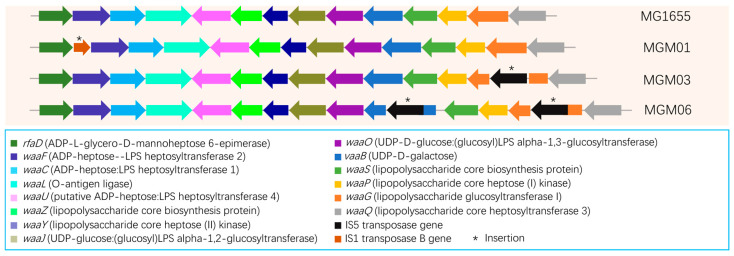
**Schematic locations of IS1 and IS5 elements in the *waa* region (formerly *rfa*) of *E. coli* PRM strains.** Genes are represented as arrows (the length of the arrows correspond to the length of the genes and the direction of the arrows represent the direction of gene). Insertion mutations are represented as asterisks. Functions of the genes with different colors are also listed in the figure.

**Figure 6:**
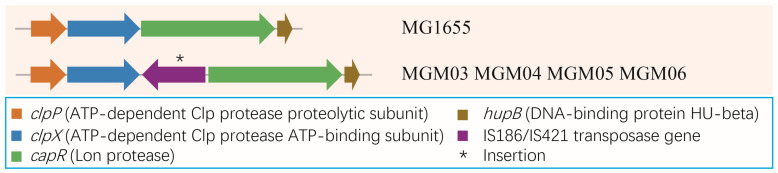
**Schematic location of the shared IS186/IS421 element in *E. coli* PRM strains.** Genes are represented as arrows (the length of the arrows corresponds to the length of the genes and the direction of the arrows represent the direction of gene). The insertion gene is shown with an asterisk. Functions of the genes with different colors are also listed in the figure.

Notably, insertions of IS elements were frequently observed in the PRM strains. A 1199 bp insertion containing a complete IS5 element at nt3806207 was found in MGM03 (Figure 5). An identical IS5 element was found to insert twice in MGM06, resulting in a breakage of *waaG* at nt3806207 and a breakage of *waaB* at nt3803912 (Figure 5). Both *waaG* and *waaB* are responsible for LPS biosynthesis. Additionally, the IS1 element transposase gene *insB9* was inserted at the very start of *waaF* (Figure 5), which destroyed the integrity of an operon including *rfaD*, *waaF*, *waaC*, and *waaL*. Bioinformatic analysis suggested that the expressions of *waaF*, *waaC*, and *waaL* were likely eliminated. Moreover, MGM03–MGM06 shared the same insertion of the IS186/IS421 transposase gene *insL* at nt456799 (Figure 6), which is very close to a small RNA gene *sraA*, and the transcriptional start site of the gene *capR* coding for a Lon protease CapR. Hence, the insertion of IS186/IS421 implies a potential effect on the expression levels of SraA and CapR in the bacterium.

### 3.6. Genomic Resequencing Reveal Divergent Evolutionary of Coevolved E. coli Populations

**Figure 7:**
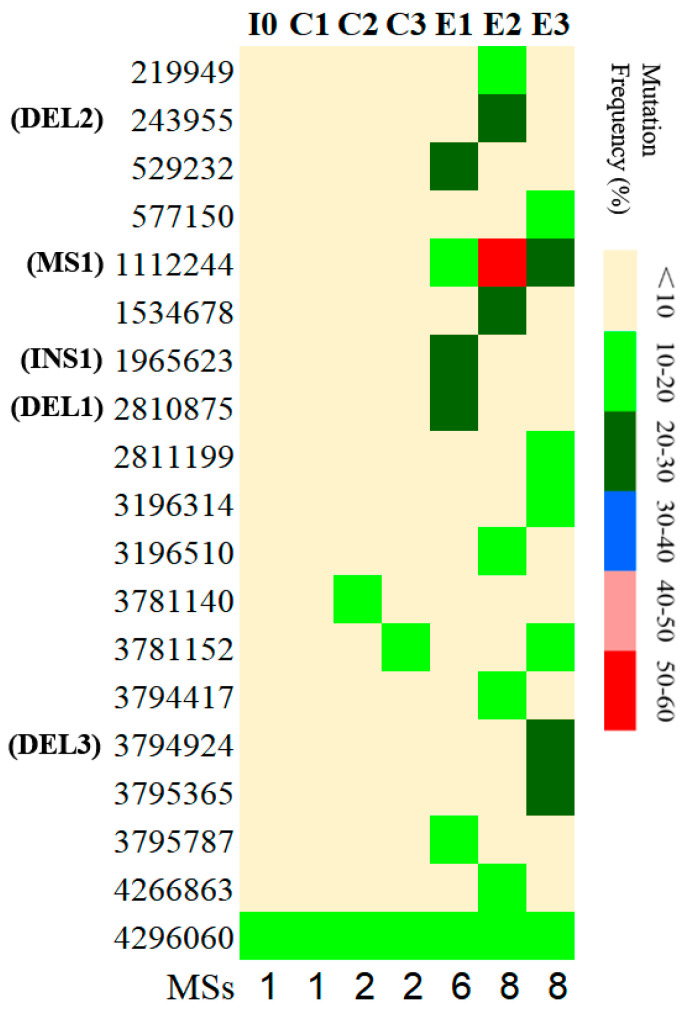
**The distribution and mutation frequencies of MSs in the evolved and coevolved *E. coli* cell populations revealed using resequencing.** I0: the collected sample before grouping at the early exponential stage (OD_600nm_ = 0.6–1); C1: collected samples from evolved cell populations at 21 h; C2: collected samples from evolved cell populations at 27 h; C3: collected samples from evolved cell populations at 33 h; E1: collected samples from coevolved cell populations at 21 h; E2: collected samples from coevolved cell populations at 27 h; E3: collected samples from coevolved cell populations at 33 h.

The MG1655 cells that grew normally without phages were set as evolved *E. coli* populations, while the cells that grew together with enriched and mixed phages were set as coevolved *E. coli* populations. To determine the MSs and their occurrence frequencies in the evolved and coevolved *E. coli* populations, they were resequenced at different time points; in total, 19 MSs were discovered (Table 2). There were no detectable MSs in the evolved populations except for three MSs at nt3781140, nt3781152, and nt4296060 (Figure 7). The MS at nt3781140 with a mutation frequency of 11% only merged in the C2 group. The MS at nt3781152 merged in the C3 and E3 groups with rates of 11% and 10%, respectively. The MS at nt4296060 was shared in all the groups. The mutational frequencies of this site in all groups remained relatively low (14.0–17.5%) and no obvious differences in mutational frequencies in the evolved and coevolved populations were observed. Both MSs (at nt3781140 and nt3781152) occurred in non-coding and non-promoter regions, and thus, they probably had no effect on the phenotype. Comparatively, there were 16 unique MSs in the coevolved cell populations of groups E1, E2, and E3, accounting for 84% of the MSs detected, among which eight MSs were identified as InDels (Figure 7). The cell populations in the coevolved group E3 had a big deletion fragment (DEL3) containing at least 10 bases (at nt3794924). These results clearly demonstrate that coexisting phages raised the mutation rates and expedited the emergence of detectable MSs in the *E. coli* cell populations.

Completely different distribution of MSs in the three coevolved populations at different periods were observed (Figure 7). In the E1 group, six SMs were generated, among which the DEL1 site represented a unique DNA deletion containing 11 bases in the gene *mprA*. Moreover, the E1 group harbored a unique 68 bp insertion site INS1, a segment of *insB* from the IS1 element, which was inserted into the gene *flhB*. In the E2 group, eight SMs were generated, among which the DEL2 site represented a unique deletion containing 10 bases in the gene *gmhA*. In the E3 group, eight SMs were generated, among which the DEL3 site also represented a 16-base unique deletion across the gene *waaF* and its proximate promoter region. These results indicate that the genetic compositions of the *E. coli* cell populations that coevolved with enriched and mixed phages changed dynamically, and the existence of infection pressure by phages drove and accelerated the divergent evolution of the *E. coli* cell populations.

### 3.7. MS-Associated Genes in Coevolved Cells and Their Predicted Functions

The MS-associated genes and their functions are listed in Table 2. Most MS-associated genes were found to be involved in the biogenesis of polysaccharides, LPS, and the cell membrane/envelope. Here, we focused on point mutation MS1 at nt1112244 and three deletions—DEL1, DEL2, and DEL3 (Figure 7, Table 2)—as all of them caused premature transcription termination. MS1 was the only MS shared by the three coevolved cell populations that was not detected in the evolved cell populations (Figure 7). The substitution from G to A at MS1 occurred with a high average frequency of 30.2% in the coevolved *E. coli* cell populations and caused a stop-gain in the middle of the gene *opgH*. The protein OpgH (also known as MdoA) encoded by *opgH* is a glycosyltransferase involved in the synthesis of osmoregulated periplasmic glucans. In the E1 group, the deletion of DEL1 generated a truncated edition of gene *mprA* coding for the multiple-transcription repressor protein, which resulted in a predicted 46 aa deletion at the N-terminus of MprA. Furthermore, the E1 group contained a *unique insertion* site INS1, causing an unexpected transcription termination in the middle of the gene *flhB*, which encodes a flagellar biosynthesis protein.

The deletion of DEL2 in the E2 group generated a truncated edition of the gene *gmhA*. In *E. coli*, *gmhA*, *gmhB*, *hldD*, *hldE*, *rfaC*, *rfaF*, *rfaH*, and *rfaP* are involved in the synthesis of the inner core of lipopolysaccharide (LPS) [39]. The deletion site DEL3 in the E3 group included 11 bases at the upstream and 5 bases at the start of the gene *waaF*. This predictably resulted in a breakage of the *waaDFCL* operon in the *waa* region that contains the major core–oligosaccharide assembly operons in *E. coli* [40], and the downstream genes, including *waaF*, *waaC*, and *waaL*, could not be normally transcribed.

## 4. Discussion

Genetic variation research on bacterial evolution can elucidate evolutionary mechanisms and uncover novel connections between genotypes and phenotypes. MG1655 is one of the most widely used *E. coli* strains in fundamental microbial studies and is also the parental strain of widely used engineering *E. coli* strains [41]. The complete genome of *Escherichia coli* MG1655 is also the standard reference genome. Therefore, we used this strain and its genome for all the experiments and analysis. In this study, 101 PRM strains of *E. coli* were purified from resistant mutant colonies, and there is a possibility that all these PRM strains may harbor interesting and novel genetic variations. However, it is hard to analyze all the strains in a limited space and may have detracted from our main goal. IRS-PCR is a simple and reliable method for differentiating conspecific strains with genetic diversity [29,42,43], and only six PRM strains exhibited obvious genetic variations using IRS-PCR. Therefore, we utilized these six PRM strains to further explore the genetic variations, as they were most likely to harbor significant genetic mutations. In addition, bacterial cells naturally face more complex environments that are inundated with various phages, and therefore we adopted mixed phages to simulate a natural environment wherein bacterial cells endure harsher phage infection pressure.

Mobile genetic elements (MGEs) are DNA elements that can move relatively freely to other locations in the host DNA, including IS elements, transposons, and gene cassettes/integrons, as well as plasmids and integrated junction elements [44,45]. MGEs promote horizontal genetic exchange and play a key role in the environmental adaption, genetic diversity, and genomic evolution of microbes [46]. IS elements are the simplest MGEs and are ubiquitous in bacterial genomes. They only encode a transposase flanked by distinctive short inverted repeats that set the element apart from the host DNA [47]. In this study, the six PRM strains harbored at least eight replicative transpositions of IS elements, including four insertions of IS186/IS421 (50%), three insertions of IS5 (37%), and one insertion of IS1 (13%) in the limited gap sites that were amplified and sequenced. Coincidentally, the IS1 element was found to be inserted in the gene *flhB* at a frequency of 21% in the coevolved cell population based on genome resequencing analysis.

Previous studies have found some insertion hotspots of IS elements and their regulation of gene expression in hosts under stress [47]. Early studies indicated that gene transcription can be enhanced by IS5 when it is placed on either side of the promoter of a target gene in *E. coli* [48]. The first and best-studied example demonstrated that an IS5 element was involved in the regulation of the glycerol utilization operon *glpFK* in *E. coli* through insertion into a specific activating site upstream of the operon [49]. This event promotes high-level *glpFK* operon expression, allowing for glycerol utilization in wild-type cells under inhibitory conditions [49]. However, the IS5 element in our findings may play a negative role, as it was inserted in the gene-coding region of two closely related genes, namely, *waaB* and *waaG*, in the *waa* (formerly *rfa*) operon. WaaG encoded by *waaG* is a retaining glycosyltransferase that anchors to the bacterial inner membrane [50], and in *E. coli*, it has been associated with biofilm degradation and flagellar synthesis [51]. WaaB is another core glycosyltransferase and is involved in the assembly of the outer core of LPS; a mutation in *waaB* leads to a truncated lipid A core [52]. A previous study revealed that in a phage-resistant *E. coli* strain, aberration in the *rfa* gene cluster caused the insertion of an IS5 element; this mediated a deletion that encompassed the *rfaBIJ* genes in *E. coli,* which was responsible for the phage resistance of a mutated strain. Complementation analysis showed that the product of *rfaB* (now *waaB*) is necessary for the synthesis of LPS, which serves as a co-receptor for bacteriophage K20 binding [53]. In our study, frequent insertions of IS1 and IS5 elements in the *waa* region were also found, and it predictably destroyed the normal synthesis of LPS. These results strengthen our speculation that the *waa* region responsible for LPS synthesis is the hotspot for the hopping of IS elements in *E. coli* under phage infection pressure, and the regulation of *waa* region by the insertion of IS elements is probably a common strategy for *E. coli* to cope with phage infection stress. Another insertion by the IS186/IS421 element at the promoter of *capR* was found in four out of the six PRM strains, though there is no direct correlation between *capR* regulation and phage resistance. The role of *capR* regulation by the insertion of IS186/IS421 in the PRM strains needs to be deeply explored in future studies.

Inactivating or enhancing expression via the insertion of IS elements is not the only way that IS elements regulate bacterial gene expression. In this study, gene deletions, including the *flhDC* operon adjacent to an IS1 element, were also observed in a PRM *E. coli* strain MGM03, where the *flhDC* operon encodes transcription factors that initiate flagellar synthesis [54]. Since bacteria flagella are also common receptors of phages [55], flhDC operon deletion and interruption of a key gene *flhB* for flagella synthesis predictably had a critical contribution to the phage resistance of the PRM strains and the occurrence of coevolved cell populations. The existence of IS elements increases bacterial genome instability, including adjacent deletions, in which DNA connected to one end of the IS element is deleted without affecting the element itself. Through large-scale deletions, duplications, insertions, and chromosomal rearrangements, IS elements contribute to bacterial genetic diversity and genomic evolution [56]. Overall, the insertions of IS elements in key gene locations were frequently found in the PRM strains and coevolved cell populations; these results reveal for the first time that IS elements play a vital role in generating genetic divergence and regulating gene functions under phage infection stress.

In addition to mutation preference/bias in the genes coding for LPS, flagella, and the translocation of the IS element, some mutations that appeared to have no significant association with phage resistance had been identified in the PRM strains and coevolved cell populations, such as the genes coding for the cold shock protein YdfK and the arylamine N-acetyltransferase NhoA. Related to the abundant biochemical phenotypes of mutant strains, it is speculated that these genetic variations may be associated with the phenotypic changes in bacteria, or these alerted sites may be under selection and encoded some function important for adaption to the abiotic environment [21].

The frequent expansion and contraction of TRs was another obvious feature observed in the PRM strains. TRs are common and extremely unstable genomic components, originally known as one of the ‘selfish’ junk DNA [57]. However, increasingly more studies have shown that TRs can enrich phenotypic changes through mutations in the coding region, promoter region, or another regulatory region [52,53,54]. Under a stress condition, TRs can accelerate the evolution of coding and regulatory sequences and promote adaptability through the regulation of gene expression [58,59]. Therefore, we speculated that the changes in TRs in the *E. coli* PRM strains may strengthen certain adaptation, though TR-related genes theoretically have no direct contribution to their resistance to phages.

rRNA genes perform the most fundamental and important functions in life, so they are unlikely to generate rapid and frequent changes. Therefore, the rRNA genes were once considered the ideal chronometer to record the evolutionary history of life [60,61], and rRNA sequence comparisons led to the construction of a ‘universal tree of life’, dividing all life on Earth into three equidistant domains: Eukarya, Bacteria, and Archaea [62]. In addition, few mutations in tRNA genes or related DNA regions were reported in bacteria. However, it is surprising that frequent mutations of rRNA and tRNA genes and their related DNA regions were observed in the six PRM strains. Until now, there is no evidence indicating direct correlation between phage-resistant mechanisms and rRNA gene mutations. Hence, we do not know the biological significance of rRNA, tRNA, and their related mutations in bacteria under phage infection stress. The rRNA- and tRNA-related mutations in the PRM *E. coli* strains under experimental conditions urged us to speculate that similar incidents may occur with certain frequencies in natural bacterial communities due to the prevalence and strong selective stress of phages. Therefore, there is a possibility that phage infection stress can change the sequences of highly conserved rRNA genes and drive bacterial rRNA molecular evolution.

In this study, many genetic variations were observed in the isolated *E. coli* PRM strains and coevolved *E. coli* cell populations. Here, we only focused on certain genes that are predicted to be directly or indirectly responsible for the antagonism to phage invasion. *OpgH* mutation was observed in three PRM strains and in coevolved *E. coli* cell populations. OpgH is involved in the synthesis of osmoregulated periplasmic glucans, and an *opgH* mutant of *E. coli* exhibits resistance to bacteriophage MS2 [63]. This suggests that *opgH* mutation is probably critical for three PRM strains against enriched and mixed phages and for the persistent existence of phage-resistant subpopulations during different periods. MprA is a multiple-transcription repressor protein in *E. coli*, where early studies showed that it negatively regulates the biosynthesis of the peptide microcin [64] and the expression of the multidrug resistance pump EmrAB [65]. Yang et al. (2004) [66] found that overproduction of MprA from a multicopy plasmid caused the occurrence of multidrug resistance, and mutator phenotypes contained a large increase in frameshift and base substitution mutagenesis, which indicates that *mprA* is a mutator gene. MprA contains a ligand-binding domain located in the N-terminal domain; therefore, the destruction of the N-terminal domain predictably increases the activity of mutant MprA [66]. As such, the DEL1 mutation of *mprA* probably increases the genomic instability and exerts a far-reaching impact on various MSs in coevolved *E. coli* populations. Assuming this to be the case, how phage infection stress is transmitted to *mprA* is another interesting question. Bacterial flagella and LPS often serve as receptors in a phage invasion and are the first defense for bacterial hosts [67,68]. Bacterial hosts frequently change their LPS and flagella structures to avoid phage infections by generating gene mutations involved in flagella and LPS synthesis [69]. Thus, it is no surprise that various genes (such as *flhB*, *gmhA*, *waaG*, *waaB*, and *waaF*) involved in flagella and LPS synthesis contain many MSs in the PRM strains and coevolved populations. However, it is notable that the PRM strains exhibited genetic diversity via IRS-PCR and genome sequencing, and the profiles of MSs in coevolved cell populations with enriched and mixed phages were very different at three coevolved stages (E1, E2, and E3), wherein the coevolved populations contained the most defective MSs. This suggests that *E. coli* host cells at different coevolving stages adopt different strategies to withstand phage infection and that phage infection stress accelerates the occurrence of detectable bacterial mutants. Pal found that after fewer than 200 bacterial generations, 25% of *Pseudomonas fluorescens* populations coevolving with phages presented 10- to 100-fold increases in mutation rates owing to mutations in mismatch repair genes, while the populations evolving in the absence of phages showed no significant change in the mutation rate [22]. Actually, the infection and anti-infection mechanisms persisting between phages and their hosts lead to a phage–host arms race [70], which is believed to be one of the driving forces shaping bacterial community evolution [70,71].

Numerous genetic variations in *E. coli* PRM strains and coevolved populations also raised the question of whether phage infection stress induced the generation of most of the observed mutations in *E. coli*. It was estimated using whole-genome sequencing that spontaneous mutations in *E. coli* occur at a rate of 1.0 × 10^−3^ mutations per genome per generation [72]. According to this spontaneous mutation rate, it is impossible to accumulate numerous genetic variations in the genomes of *E. coli* PRM strains and coevolved populations, even over the longest generation time of 33 h (about 156 generations) for the E3 group. Therefore, the increased genetic variations (mutations) in *E. coli* PRM strains and coevolved populations were not generated by spontaneous mutations but mainly induced by phage infection stress, which provides one important force driving the genomic evolution of *E. coli* and accelerates the genetic divergence via an increased mutation rate. Swings found that mutation rates of *E. coli* populations increase when cells experience higher stress and decrease again once cells are adapted, and they identified cellular mortality as the major force driving the quick evolution of mutation rates [73]. In some cases, environmental stress can increase the mutation rate [71,72,73]. These findings collectively provide strong evidence that mutation rate variability is a crucial factor in adaptive evolution under selective pressure [74]. The study suggests that the role of environmental stress is not limited to naturally selecting preexisting mutants and that not all mutations are random and evolution is slow. Therefore, it highlights the importance of microbial genomic evolution.

## 5. Conclusions

The *E. coli* PRM strains exhibited abundant genetic and phenotypic diversity. Resequencing of evolved and coevolved *E. coli* populations revealed that the coevolved populations at three evolving stages had discrepant profiles of MSs while nearly all detected MSs occurred in the coevolved populations. IS elements played a vital role in creating various genetic divergences and regulating gene functions under phage infection stress. Frequent expansion and contraction of TRs was another obvious feature observed in the PRM strains, which likely strengthen certain adaptation of the PRM strains. Frequent mutations were observed in rRNA and tRNA genes or their related DNA regions in the PRM strains under phage infection stress, which complicate bacterial phylogenetics based on rRNA genes. Our results, combined with other findings, indicated that phage infection stress boosts mutation rates in *E. coli* genome and accelerates the occurrence of divergent *E. coli* mutants.

## Figures and Tables

**Table 1:** Scattered 12 SPM sites and one InDel in the PRM strains derived from *E. coli* MG1655.

Mutation Sites	Variation; AA Change	Gene	Protein Product	Protein Name	1	2	3	4	5	6
1050039	A > C; S > R	*insB-4*	NP_415508.1	IS1 protein InsB	√	√	√	√		
1112244	G > A; W > *	*opgH*	NP_415567.1	osmoregulated periplasmic glucans (OPGs) biosynthesis protein		√			√	√
1633223	A > N/C; K > T	*ydfK*	NP_416062.2	Qin prophage; cold shock protein YdfK		√			√	
1633583	A > N	*ydfK*	NP_416062.2	Qin prophage; cold shock protein YdfK		√				
2066465	T > C; K > R	*insH-6*	NP_416498.1	CP4-44 prophage; IS5 transposase and trans-activator						√
3801210	G > T; H > N	*waaJ*	NP_418083.1	UDP-glucose: (glucosyl) LPS alpha-1,2-glucosyltransferase				√		
3801516	G > A; Q > *	*waaJ*	NP_418083.1	UDP-glucose: (glucosyl) LPS alpha-1,2-glucosyltransferase		√				
4507837	G > C; L > V	*insI-3*	NP_418704.1	KpLE2 phage-like element; IS30 transposase					√	
1633629	T > C; Q > R	*pinQ*	NP_416063.1	Qin prophage; putative site-specific recombinase pinQ					√	
1635957	InDel	*nohA*	YP_009518788.1	Qin prophage; putative prophage DNA-packaging protein NohA					√	
580437	A > C; S > R	*ylcI*	YP_001165309.1	DLP12 prophage; DUF3950 domain-containing protein YlcI					√	
4236664	C > T; Q > *	*yjbF*	NP_418451.4	lipoprotein YjbF				√		
19933	G > A; H > Y	*insB-1*	NP_414562.1	IS1 protein InsB						√

The locations of MSs refer to the position of *E. coli* MG1655 (NC_000913.3). Indel: a 7-bp insertion, TGCACCN. 1–6 in the right columns represent PRM strains MGM01–MGM06, respectively. “√” represents one PRM strain contain one certain mutation site. “*” represents a stop codon.

**Table 2:** Mutation sites and genes with rapidly accumulated mutations in *E. coli* evolved and coevolved populations.

Position	Variation	Mutation Type	AA Change	Protein Name	Protein Function
219949	C > T	nonsynonymous SNV	C359T; R120H	Outer membrane lipoprotein (RcsF)	Polysaccharide synthesis
243955	GAATGAAAGT > -	frameshift deletion	413_422del; G138fs	D-sedoheptulose 7-phosphate isomerase (GmhA)	LPS synthesis
529232	G > A	downstream	-	DNA-binding transcriptional regulator (YlbG)	Transcription regulation
577150	G > A	upstream	-	DLP12 prophage; phage holin protein	Membrane lysis
1112244	G > A	stopgain	G1382A; W461X	Osmoregulated periplasmic glucans biosynthesis protein (OpgH)	Polysaccharide synthesis
1534678	A > T	nonsynonymous SNV	A655T; N219Y	Arylamine N-acetyltransferase (NhoA)	Acetyltransfer
1965623	Insertion of 68 bases	stopgain	568_569ins68 bases; G_190_L_191_delinsELHDKVIGHYLNIKHYQX	Flagellar biosynthesis protein (FlhB)	Flagellar biosynthesis
2810875	ATGCAAAGCAA > -	frameshift deletion	106_116del; M36fs	DNA-binding transcriptional repressor (MprA)	Multidrug resistance
2811199	- > GC	frameshift insertion	430_431insGC; S144fs	DNA-binding transcriptional repressor (MprA)	Multidrug resistance
3196314	A > -	frameshift deletion	995del; V332fs	Fused heptose 7-phosphate kinase/heptose 1-phosphate adenyltransferase (RfaE)	LPS heptosyl transfer
3196510	GC > -	frameshift deletion	243_244del; A81fs	Fused heptose 7-phosphate kinase/heptose 1-phosphate adenyltransferase (RfaE)	LPS heptosyl transfer
3781140	A > G	upstream	-	tRNA(cytidine/uridine-2\’-O)-ribose methyltransferase	tRNA methyl transfer
3781152	A > G	upstream	-	tRNA(cytidine/uridine-2\’-O)-ribose methyltransferase	tRNA methyl transfer
3794417	A > C	synonymous SNV	A429C; S143S	ADP-L-glycero-D-mannoheptose 6-epimerase (RfaD)	Core LPS precursor synthesis
3794924	TCTGCATGAAAATACT > -	frameshift deletion	Whole gene	ADP-heptose-LPS heptosyltransferase 2 (WaaF)	LPS heptosyl transfer
3795365	- > C	frameshift insertion	438insC; R146fs	ADP-heptose-LPS heptosyltransferase 2 (WaaF)	LPS heptosyl transfer
3795787	T > A	nonsynonymous SNV	T859A; Y287N	ADP-heptose-LPS heptosyltransferase 2 (waaF)	LPS heptosyl transfer
4266863	C > T	downstream	-	Alanine racemase 1	Alanine synthesis
4296060	C > T	downstream	-	Glutamate/aspartate: H (+) symporter GltP	Amino acid transport

“-” represents the absence of the base-pairs.

## Data Availability

The original contributions presented in this study are included in the article/Supplementary Materials. Further inquiries can be directed to the corresponding author.

## Supplementary Materials

The following supporting information can be downloaded at: https://www.mdpi.com/article/10.3390/microorganisms14010160/s1, Table S1: MS sites and their related genes in coevolved *E. coli* cell populations with phages (XLSX).
